# Research Progress in Interfacial Characteristics and Strengthening Mechanisms of Rare Earth Metal Oxide-Reinforced Copper Matrix Composites

**DOI:** 10.3390/ma15155350

**Published:** 2022-08-03

**Authors:** Xuemin Fu, Jiaxin Jiang, Xiaosong Jiang

**Affiliations:** 1 School of Intelligent Manufacturing and Equipment, Chengdu Textile College, Chengdu 611731, China; fuxuemin04@163.com; 2 School of Materials Science and Engineering, Southwest Jiaotong University, Chengdu 610031, China

**Keywords:** rare earth metal oxides (REMOs), copper matrix composites, interface, microstructure, mechanical properties

## Abstract

The existence of a small amount of rare earth metal oxides (REMOs) can greatly affect the structure and function of copper matrix composites owing to improvement of surface and interface properties between REMOs and metal matrix, and there are still some challenges concerning interfaces and complex interfacial reactions. This review summarizes the interfacial characteristics and strengthening mechanisms of REMO-reinforced copper matrix composites, including fabrication methods for solving rare earth metal oxide-dispersion problems and characterization of the microstructure and properties of REMO-reinforced copper matrix composites. In particular, the strengthening effects of various rare earth metal oxide-reinforced copper matrix composites are systematically summarized. The interface characteristics of composites from a thermodynamics standpoint and the strengthening mechanism are emphatically investigated and discussed in order to help unveil design principles and to provide reference for future research of REMO-reinforced copper matrix composites.

## 1. Introduction

With excellent thermal conductivity, electrical conductivity and machinability, only the mechanical properties of copper limit its wide application in electrical contact materials and wires [1]. In general, whiskers, particles, fibers and other reinforcements are used to improve the mechanical properties of copper [2]. Due to their excellent thermal, electrical and mechanical properties, copper matrix composites are widely used in the machine manufacturing industry, electronics, the military, the aerospace industry, etc. [2]. In practice, the thermal and electrical conductivity of the copper matrix is usually affected by the addition of the reinforcements [3]. With science and technology continually improving, higher requirements are put forward for the performance of copper matrix composites to ensure the coordinated improvement of electrical conductivity and mechanical properties [4]. Rare earth elements (elements or oxides) are known as “industrial vitamins” [5]. Rare earth elements are one of the good choices as reinforcement in composites. Adding the proper amount of rare earth elements cannot only refine the grain, reducing the wettability angle between reactants, but it can also change the morphology and distribution of impurities to improve the comprehensive properties of copper matrix composites [5].

Rare earth elements are widely used throughout electronic, petrochemical and other fields due to their excellent physical, chemical and electrical properties [6]. Rare earth elements are one of the most reactive metals; at room temperature, they are almost insoluble in copper, so it is easy to form compounds with other alloying elements, and they play a very important role in eliminating alloy melt contamination [7]. For example, doping with the rare earth element Sc significantly inhibits discontinuous precipitation and leads to continuous precipitation within the grain; thus, the hardness of the alloy is improved [8]. Rare earth elements are surface-active elements that can improve the fluidity of the metal matrix by reducing the surface tension of the metal matrix [9]. The decrease of the surface tension leads to increased wettability of the reinforcement in the metal matrix, which increases the surface diffusion coefficient of the reinforcement [10]. In addition, the solid solubility of rare earth metals in the metal matrix is relatively low and easily adsorbed at the phase boundary, which not only fills interface defects, but also improves the interface energy between the reinforcement particles and the metal matrix [11]. Therefore, relatively dispersed reinforcements are easy to form in the metal matrix [12]. For example, the coordination bond between Ce and the oxygen-containing group of GO reduces the surface energy of GO and improves the dispersion of GO [3]. The addition of the proper amount of rare earth elements or their compounds can improve toughness, plasticity, corrosion resistance and heat resistance [13]. Recent studies have shown that the addition of rare earth can reduce the probability of electron scattering in copper alloys and thus increase the electrical conductivity to a certain extent [14]. Rare earth elements can improve the conductivity and mechanical properties of copper alloy to a certain extent because they can purify the melt and refine the grain structure of copper alloys [4,15].

Rare earth oxides can be used as dispersion strengthening phases owing to their excellent thermodynamic stability. Rare earth oxides also have the following advantages over general metal oxides [16,17,18,19,20,21]: First, the thermal expansion coefficient (CTE) of rare earth oxides is usually higher than that of general metal oxides (for example, the CTEs of Y_2_O_3_, CeO_2_, Cr_2_O_3_ and Al_2_O_3_ are 9.3 × 10^−6^ °C^−1^, 13 × 10^−6^ °C^−1^, 8.4 × 10^−6^ °C^−1^ and 7.6 × 10^−6^ °C^−1^, respectively); when the rare earth oxide is used as the second phase to strengthen the metal matrix, this characteristic enables it to ensure the mechanical properties of the composite. Second, rare earth elements have larger atomic radii than general metals; therefore, they have lower solubility and diffusivity in the matrix, thus improving the coarsening resistance stability of the microstructure, which is crucial for the preparation of advanced multifunctional metal matrix composites with both high strength and high electrical or thermal conductivity. Third, recent studies have shown that some rare earth oxides belong to P-type conductors, and their addition to copper alloys or copper matrix composites such as Y_2_O_3_ can significantly improve the electrical properties of the composites. Fourth, rare earth oxides such as Sc_2_O_3_ can provide heterogeneous nucleation cores for the interface and the copper matrix, thus refining the grain size of the copper matrix and improving the comprehensive performance of copper matrix composites.

Based on the fact that the role and influence of rare earth elements and their oxides in copper matrix composites are closely related to their own thermodynamic properties, this paper analyzes the thermodynamic properties of rare earth elements in order to analyze their constituent elements with copper matrix composites and their mechanism between phases; this is a key factor to research the action mechanism of rare earth elements on copper matrix composites.

## 2. Influence Mechanism

### 2.1. Purifying

It is well known that rare earths (Res) can react with harmful impurities (H, O, Cl, S, Pb, Bi, etc.) to purify alloy melt, and that the purification effect comes from their own chemical properties. Compared with the substrate metal, rare earths are more likely to react with impurities or oxygen to adjust and control for the chemical composition of the matrix. At the same time, the reaction product of high melting-point-spread in copper matrix can refine the as-cast structure and improve the microstructure and properties of the composites.

**Figure 1:**
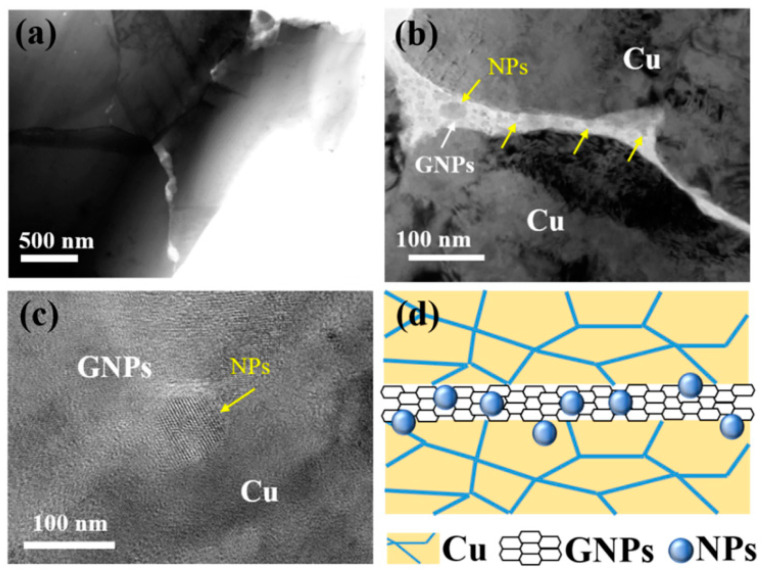
TEM and HRTEM images of existing form and dispersion of nanoparticles: (**a**) TEM of existing morphology of GNPs within the matrix; (**b**) Enlarged TEM images of (**a**); (**c**) HRTEM images of existing form of nanoparticles; (**d**) Schematic illustration of dispersion of nanoparticles [16]. Reprinted/adapted with permission from Ref. [16]. Copyright year 2020, Elsevier.

**Figure 2:**
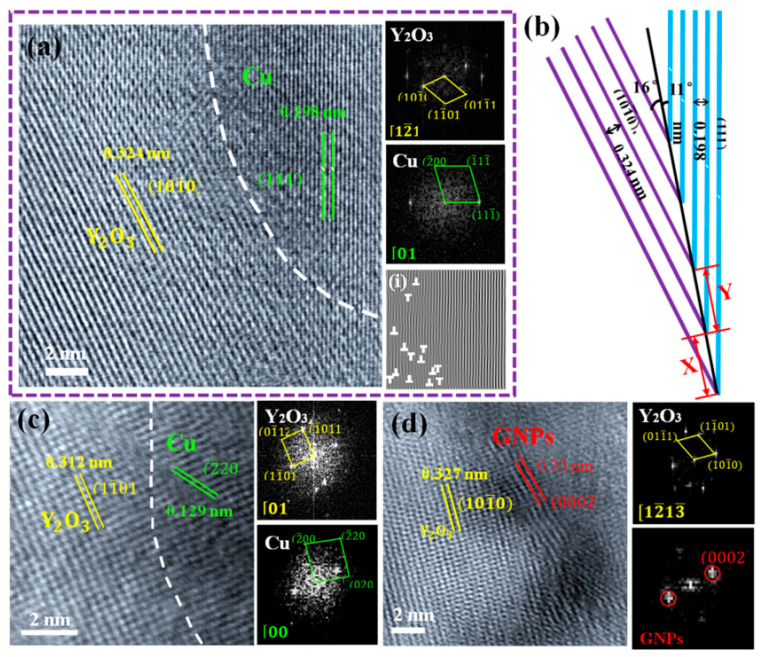
HRTEM images of the interface structure between Y_2_O_3_ and Cu and related FFT images: (**a**,**c**) HRTEM images of the interface structure between Y_2_O_3_ and Cu and related FFT images, (**i**) is the IFFT image at the interface of Y_2_O_3_ and Cu of (**a**); (**b**) Schematic diagram of the interface configuration of (**a**); (**d**) HRTEM images of the interface structure between Y_2_O_3_ and GNPs and related FFT images [16]. Reprinted/adapted with permission from Ref. [16]. Copyright year 2020, Elsevier.

**Figure 3:**
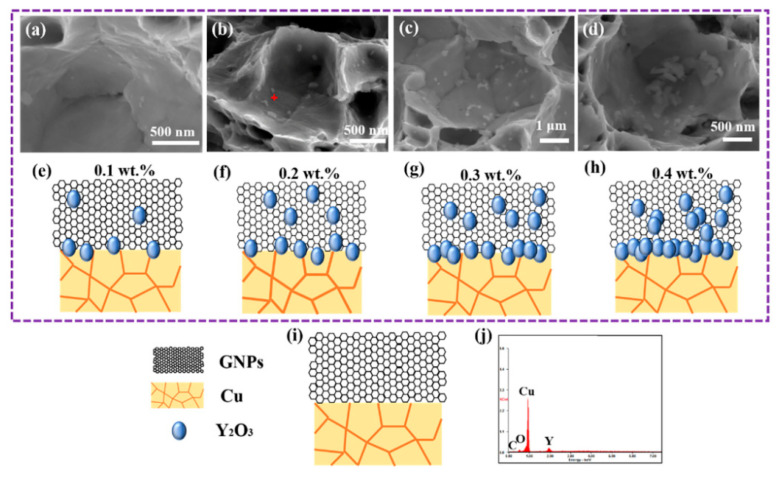
SEM images and schematics of fracture of GNP/Cu composites with different Y element content: (**a**–**d**) SEM images of fracture of 0.1 wt.% Y, 0.2 wt.% Y, 0.3 wt.% Y and 0.4 wt.% Y, respectively; (**e**–**i**) Schematic illustration of dispersion of Y_2_O_3_ NPs in 0.1 wt.% Y, 0.2 wt.% Y, 0.3 wt.% Y 0.4 wt.% Y and GNP/Cu composites, respectively; (**j**) Representative energy spectrum of NPs lied at the fracture [16]. Reprinted/adapted with permission from Ref. [16]. Copyright year 2020, Elsevier.

Qian et al. studied the atomic electron structure and interface interaction between rare earth elements (REEs) and graphene/copper by density functional theory [16]. The mechanical properties of the composites were improved by introducing Y element into the graphene nanosheet/copper (GNPs/Cu) interface, as shown in Figure 1 [16]. The introduced Y element reacts with the oxygen impurities in the matrix to generate Y_2_O_3_ nanoparticles, which are firmly embedded in GNPs and form a semi-coherent interface with the matrix. The interface formed in this way has strong interactions, as shown in Figure 2 [16]. While yield strength of the Cu–Y@GNPs composite increases by 95.4%, the elongation remains at 21% and the conductivity remains at 90% IACS. Theoretical calculation can explain that the strengthening mechanism of Cu–Y@GNPs composites is mainly caused by load transfer caused by Y_2_O_3_ NPs, as shown in Figure 3 [16]. Yang et al. found that the Ce element in a Ce–rGO/Cu composite can ensure high conductivity of the composite through its effective purification capacity on the interface [3]. Zhao et al. studied the effect of Y addition on the properties of carbon nanotubes/copper composites, and the formation of Y_2_O_3_ affected the growth and uniformity of carbon nanotubes in the copper matrix [22]. The results show that Y addition promotes the deposition of Cr to improve catalytic activity, avoids excess carbon deposition formation during carbon nanotube growth and helps to obtain a more pure Cu substrate [22]. Due to the dislocation pinning effect between Cu and Y, the dislocation is difficult to move [22]. The addition of Y improves the electrical conductivity of the composite. The combination of chromium and copper causes lattice distortion and reduces the conductivity of copper [22]. In this way, it increases the amount of Cr precipitation and reduced the solubility of Cr in Cu in order to prevent the negative effects of Cr on conductivity, eliminate impurities, purify the copper matrix, reduce the active area of the cathode and inhibit micro-electrolytic corrosion [22]. Y can also be used as a barrier to prevent further corrosion of the composites so that the overall performance of the composite material is significantly improved [22]. Corrosion resistance and tensile strength increased by 53.28% and 35.21% respectively. The electrical conductivity increased to 90.9% of the international annealed copper standard (IACS), and the friction coefficient was reduced to 0.3 for the composite sliding against stainless steel with a 10 N load and 0.03 m/s sliding speed [22].

Cao et al. added La to TiB_2_(–TiB)Cu composite; due to the purification of the La element, the impurities in the copper melt were reduced, thereby improving its conductivity [23]. Zou et al. found that the purification of rare earth La in Cu–TiB_2_ composite helps to remove excess solution atoms, thus significantly improving the conductivity of the composite [24]. Lee et al. studied the influence of Dy addition on the electrical properties and microstructure of annealed Cu–Mn alloy film and found that Dy addition can inhibit the formation of MnO and improve the resistivity of Cu–Mn film [25]. Zhang et al. studied the influence of Ce on the microstructure and mechanical properties of pure copper and found that the addition of trace Ce purified the grain interface, refined the structure, removed impurities and slag, and thus improved the ductility of pure copper [26]. However, with increased Ce addition, Ce is not only consumed in the slagging process but also reacts with the substrates Cu, P and another unremoved impurity to form a large number of intergranular and ingranular second phases and reduces the elongation of pure copper [26]. Zhang et al. found that an appropriate amount of La could make Al_2_O_3_ evenly distributed [27]. The results show that tensile strength, yield strength and elongation of Cu–Al_2_O_3_–La were improved greatly [27]. There is a semi-coherent interface between Cu and Al_2_O_3_, and the interface energy is very low. The addition of La changes the shape of Cu grains into irregular bands. This change leads to a decrease of grain boundary density, which reduces the electrical resistance. Elemental La can exist as La_2_O_3_ [27].

In summary, due to the unique purification properties of rare earth elements, the second phase can be generated by reaction with the matrix and dispersed evenly into the matrix. In addition to regulating the chemical composition of the matrix material, it can also inhibit the generation of harmful compounds and refine the crystal structure of the matrix, thus improving the overall performance of copper matrix composites.

### 2.2. Grain Refinement

The grain refining effect of rare earth elements in copper matrix composites comes from their natural properties. In general, rare earth elements will become the second phase in the composites with different chemical properties from the matrix, such as elemental and oxide. The second phase in the matrix can control the uniformity of microstructure and significantly hinder the movement of the grain boundary and grain growth to obtain a composite with fine grain size.

**Figure 4:**
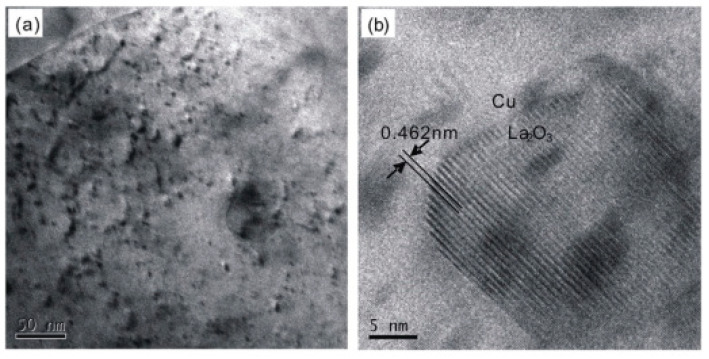
TEM images of Cu–1.3 wt.% La_2_O_3_ composite: (**a**) bright field image; (**b**) high resolution TEM image [17]. Reprinted/adapted with permission from Ref. [17]. Copyright year 2019, Elsevier.

Zheng et al. studied a Cu–La_2_O_3_ composite material and found that particles were uniformly dispersed in the copper matrix with particle size below 50 nm [17]. La_2_O_3_ generated in situ formed a good interface with the copper matrix, as shown in Figure 4 [17]. In addition, La_2_O_3_ reinforcement effectively improved microhardness and tensile strength of the composites. When La_2_O_3_ content was 1.3 wt.%, the maximum microhardness and tensile strength of Cu–La_2_O_3_ reach 118 Hv and 329 MPa, respectively [17]. Dang et al. added La_2_O_3_ to an Al–CuO system to prepare a Cu matrix composite; the size and morphology of Al_2_O_3_ particles changed significantly [5]. The size of Al_2_O_3_ particles decreased to nanometer level, the morphology changed from spherical to irregular shapes, and Al_2_O_3_ particles are uniformly dispersed in copper matrix [5]. The grains of the composites were finer and more uniform, and the conductivity and hardness were obviously improved. When 0.6% La_2_O_3_ was added to the matrix, the hardness of the composite reached 87.9 Hv, and the conductivity of the material reached 90.2% IACS [5]. Compared with the composites prepared by an Al–CuO system, the hardness and the conductivity of the composites prepared by the La_2_O_3_–Al–CuO system increased by 8.7% and 7.8%, respectively [5]. After 0.6 wt.% La_2_O_3_ was added, a large number of nano-Al_2_O_3_ particles were formed and dispersed in the matrix, which reduced the probability of electron scattering and improved the conductivity of the composite [5]. In addition, shortening of the distance between the nano-Al_2_O_3_ particles led to Orowan strengthening, greatly enhanced the dispersion strengthening, enhanced the grain-refining-agent strengthening, and the mechanical properties of the composites were significantly improved due to the combined action [5].

Qin et al. prepared Cu–Y_2_O_3_ composites by solid–liquid doping, calcination reduction and spark plasma sintering [28]. Huang et al. prepared Cu–Y_2_O_3_ nanocomposites with 10% volume fraction using CuO and Y_2_O_3_ as raw materials [29]. The results showed that, due to the pinning effect of uniformly dispersed Y_2_O_3_ nanoparticles in copper sheet, the Cu grain was obviously refined. With the increase of Y_2_O_3_ content, the distribution of yttrium oxide changed from a dispersed state to an agglomerated state, and the grain size decreased gradually [29]. This is because Y_2_O_3_ has good thermal stability—in the sintering process it will not grow up—and at the same time it can pin copper grains to inhibit copper grain growth, encouraging grain refinement. The second phase Y_2_O_3_ is distributed in the inner or intergranular region of copper grains, so it has strong hindrance to dislocation motion [29]. Joshi et al. prepared Cu–Y_2_O_3_ nanocomposites and found that Y_2_O_3_ particles distributed in the copper matrix can effectively inhibit the growth of grain, and Y_2_O_3_ particles play an important role in improving grain refinement and thermal stability [30]. Maharana et al. found that, compared with pure Cu coating, Cu–Y_2_O_3_ composite coating has significantly refined grains and better mechanical properties (wear resistance and hardness), as Y_2_O_3_ particles can improve grain refinement and thermal stability [31]. In the study of composite materials, refining grain is the main reinforcement method, mainly because grain boundaries are an important barrier to sliding in the early stage of material deformation. Thus, finely grained materials are stronger than coarsely grained materials. This dependence can be explained by dislocation accumulation at grain boundaries. The number of dislocations in these accumulations is proportional to the grain size. Thus, the stress concentration in the grains crossing the boundary increases the number of dislocations in the stacking and thus increases the grain size. Therefore, when the grain size is smaller, the applied stress required for sliding is larger, which leads to strain hardening, and the value of the strain hardening index decreases correspondingly, and vice versa. Therefore, with decreased grain size, the overall performance of a composite material is improved.

**Figure 5:**
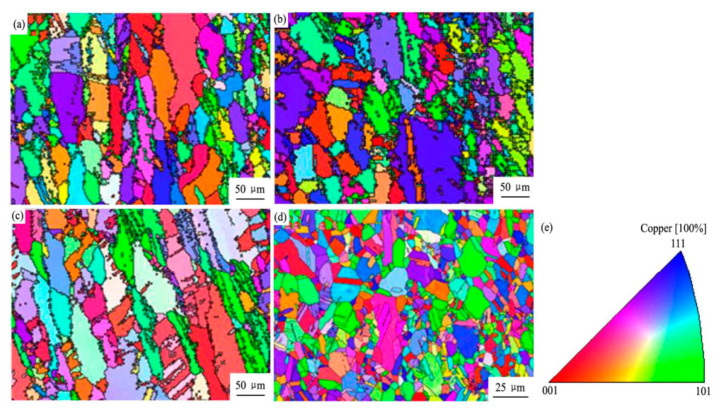
EBSD IPF maps of annealed Cu-Ag-RE samples with different Ce concentrations: (**a**) 0.05 wt.%; (**b**) 0.1 wt.%; (**c**) 0.2 wt.%; (**d**) 1.0 wt.%; (**e**) IPF coloring [15]. Reprinted/adapted with permission from Ref. [15]. Copyright year 2017, Elsevier.

**Figure 6:**
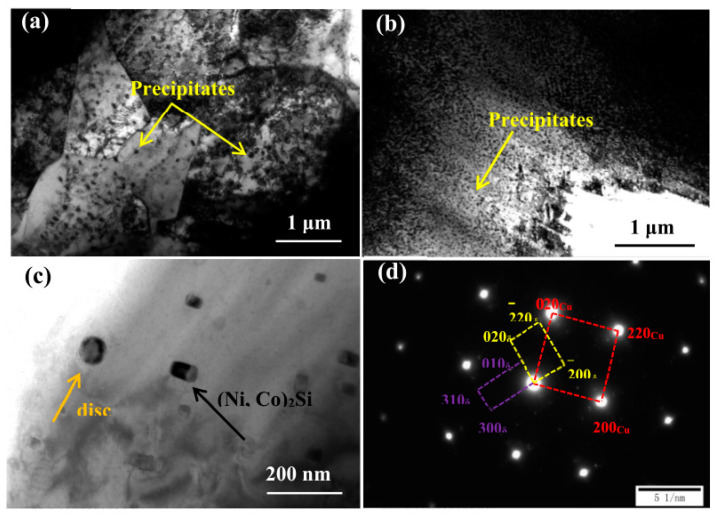
TEM images of samples deformed at 700 °C and 0.01 s^−1^: (**a**) Cu–Ni–Co–Si–Cr; (**b**,**c**) Cu–Ni–Co–Si–Cr–Ce; (**d**) SADP of corresponding area in (**c**) [13]. Reprinted/adapted with permission from Ref. [13]. Copyright year 2020, MDPI AG.

**Figure 7:**
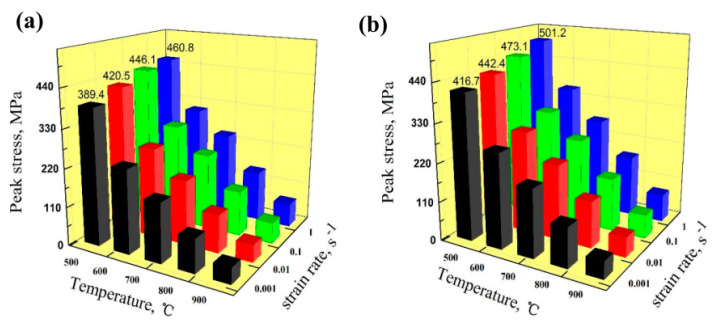
Peak stress of the Cu–Ni–Co–Si–Cr and Cu–Ni–Co–Si–Cr–Ce alloys deformed at different strain rates and temperatures: (**a**) Cu–Ni–Co–Si–Cr alloy; (**b**) Cu–Ni–Co–Si–Cr–Ce alloy [13]. Reprinted/adapted with permission from Ref. [13]. Copyright year 2020, MDPI AG.

Chang et al. found that the area of the medium axial crystal zone of as-cast Cu–Ag–Re alloy increased with increased Ce content, presenting a columnar transition to equiaxial crystal [15]. The grain structure of cold-rolled Cu–Ag–Re alloy exhibits partial or complete recrystallization with increased Ce concentration [15]. With increased Ce content, the average grain size decreases, as shown in Figure 5 [15]. Ban et al. studied the influence of elemental Ce on the microstructure and properties of Cu–Ni–Co–Si–Cr alloy [13]. As shown in Figure 6, the average grain size of Cu–Ni–Co–Si–Cr–Ce alloy is 48 μm, which is smaller than the 80 μm of Cu–Ni–Co–Si–Cr alloy [13]. The average size of the precipitated phase in Cu–Ni–Co–Si–Cr alloy is 73 nm, and in Cu–Ni–Co–Si–Cr–Ce alloy it is 27 nm [13]. The addition of Ce delayed the occurrence of dynamic recrystallization, resulting in significant improvement in the mechanical properties of the composites, as shown in Figure 7 [13]. Saha et al. found that adding elemental Sc to Cu–Zn–Al shape-memory alloy can be an effective grain refiner to produce finer precipitates and improve the hardness of the alloy [32].

**Figure 8:**
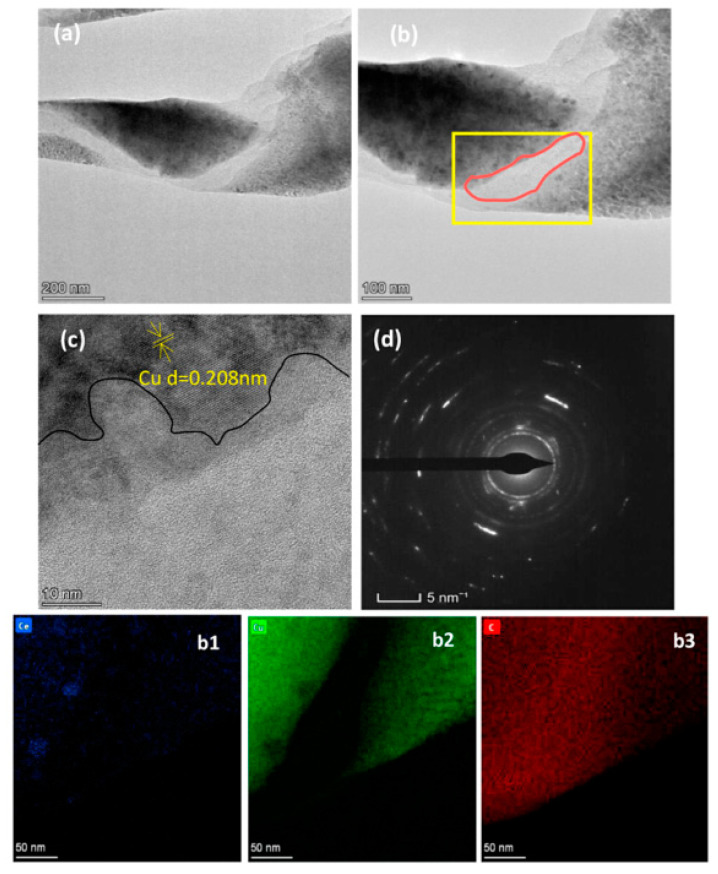
TEM, HRTEM images and EDS-mapping of Ce–rGO/Cu composite: (**a**) Low magnification TEM of Ce-rGO/Cu composite; (**b**) High magnification TEM; (**c**) HRTEM of yellow square in (**b**); (**d**) SAED patterns in red circle in (**b**) for Ce-rGO/Cu composite; EDS-mapping of (**b1**) Ce element; (**b2**) Cu element; (**b3**) C element of which pattern [3]. Reprinted/adapted with permission from Ref. [3]. Copyright year 2021, Elsevier.

**Figure 9:**
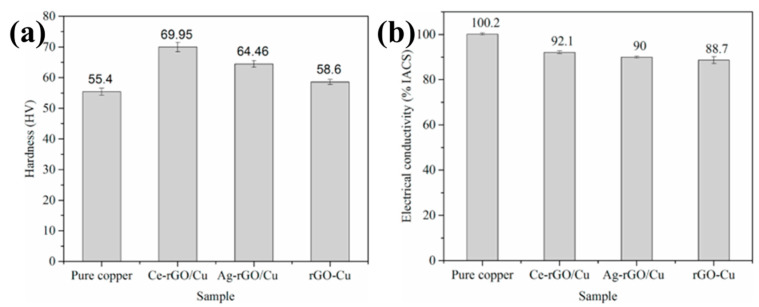
(**a**) Hardness values of graphene copper matrix composites in this study; (**b**) Conductivity of copper and different rGO-Cu composite materials [3]. Reprinted/adapted with permission from Ref. [3]. Copyright year 2021, Elsevier.

Yang et al. studied effect of Ce-modified reduction graphene oxide (rGO) on the mechanical and electrical properties of copper matrix composites [3]. The interfaces between Ce–RGO and the copper matrix are well-bonded, as shown in Figure 8 [3]. The hardness of Ce–RGO/Cu composite is 26.3% higher than that of sintered copper and 19.4% higher than that of unmodified rGO/Cu composite, as shown in Figure 9 [3]. Ce–rGO/Cu composites maintain good electrical conductivity (92.1% IACS) and good ductility (26.3% elongation) [3]. The main reason for the improved hardness of modified rGOs is the grain refinement of the matrix, and the main reason for the reduced tensile strength is the defects of modified rGOs and poor combination with the copper matrix [3]. Zhang et al. discussed the influence of rare earth oxide Sc_2_O_3_ addition to copper/diamond composites and found that it refined the microstructure of the matrix [18]. Lv et al. studied the influence of elemental La on the microstructure and properties of Cu/Ti_3_SiC_2_/C nanocomposites [33]. They found that La has a refining effect on the matrix grain, and the matrix grain size decreases with increased La content [33]. With increased La content, the properties of the composites showed a trend of first increasing and then decreasing. The composites with 0.1 wt.% La showed the best properties, with tensile strength of 174.9 Mpa and shear strength and compressive strength of 102.1 Mpa and 461.1 MPa, respectively [33]. Because dimples were observed, the tensile fracture indicates the fracture mode is ductile fracture. The enhancement mechanism of La mainly includes dispersion strengthening and fine-grain strengthening [33]. Shu et al. prepared multi-walled carbon nanotubes (MWCNTs) and graphene co-reinforced Cu–graphite composites with La by powder metallurgy and evaluated their mechanical properties [34]. Mechanical alloying and freeze-drying enhanced the interfacial bonding and particle distribution uniformity of MWCNTs or graphene-to-copper matrices [34]. The results indicated that La was diffused into the matrix to form a solid solution or compound [34]. The microstructure and mechanical properties of the composites show that the interface between the matrix and the reinforcement is closely bonded, indicating that the interface bond between the composites and the graphene–copper matrix mainly exists in the form of mechanical lock [34]. The strengthening mechanisms of the composites are mainly fine-grain, load-transfer and solution strengthening, followed by dislocation strengthening as La was dissolved in the matrix [34].

**Figure 10:**
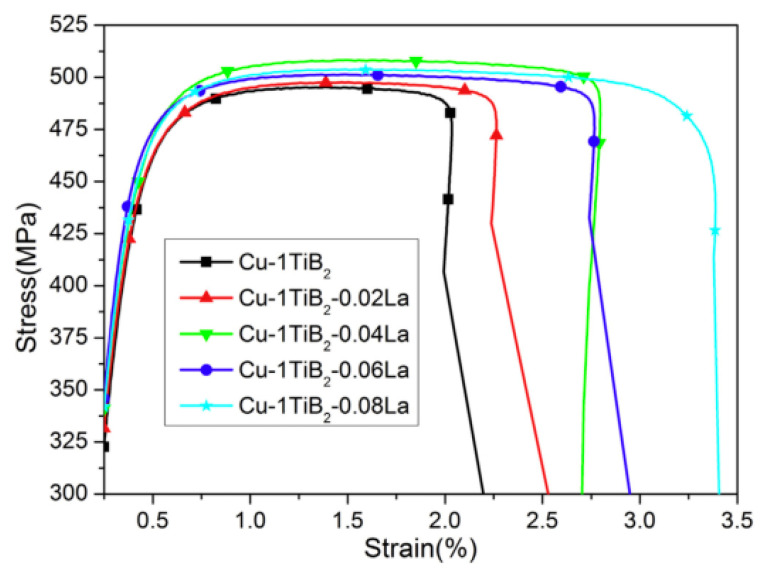
Stress–strain curves of Cu–1 wt.% TiB_2_ with different content of La [24]. Reprinted/adapted with permission from Ref. [24]. Copyright year 2016, Elsevier.

**Figure 11:**
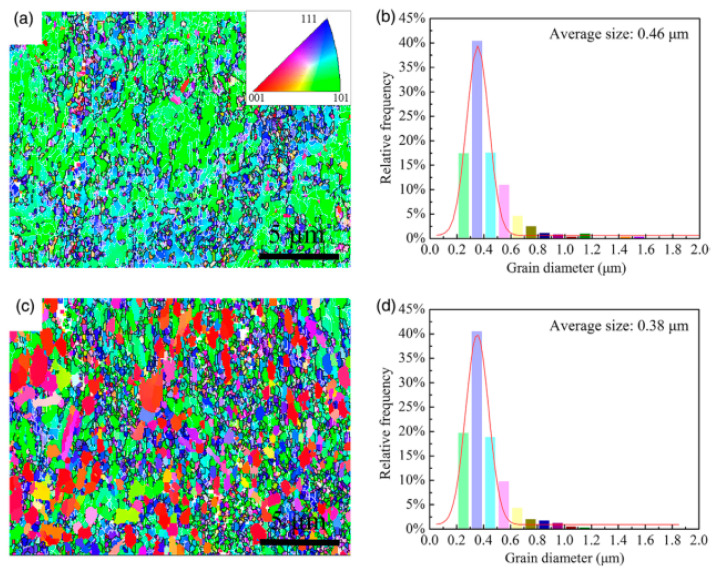
EBSD IPF maps and copper grain size distribution of as-cast (TiB_2p_ + TiB_w_)/Cu composites: (**a**,**b**) without rare-earth Ce; (**c**,**d**) with 0.04 wt.% Ce [35]. Reprinted/adapted with permission from Ref. [35]. Copyright year 2022, John Wiley and Sons.

**Figure 12:**
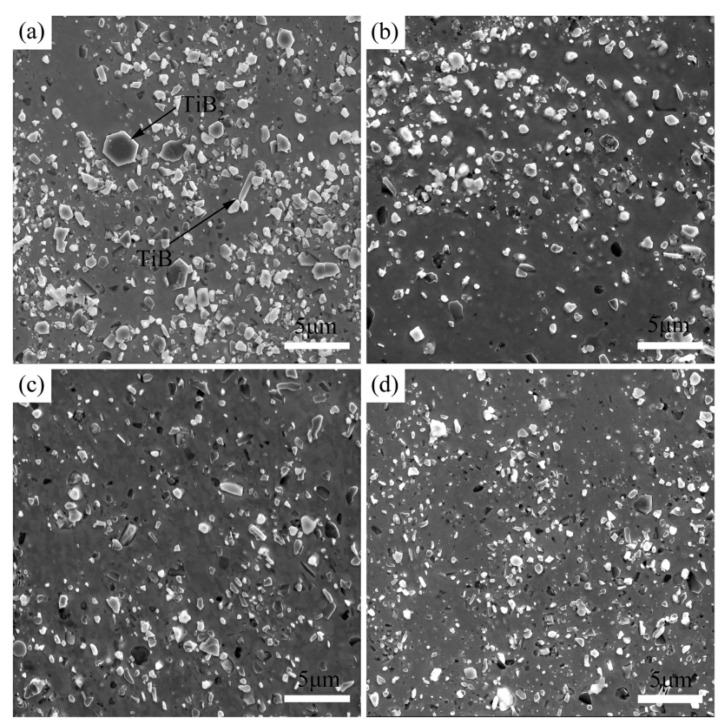
Effects of different rare earths on the microstructures of (TiB_2p_ + TiB_w_)/Cu composites: (**a**) without rare earth; (**b**) 0.04 wt.% La; (**c**) 0.04 wt.% Ce; and (**d**) 0.04 wt.% Y [36]. Reprinted/adapted with permission from Ref. [36]. Copyright year 2022, Elsevier.

**Figure 13:**
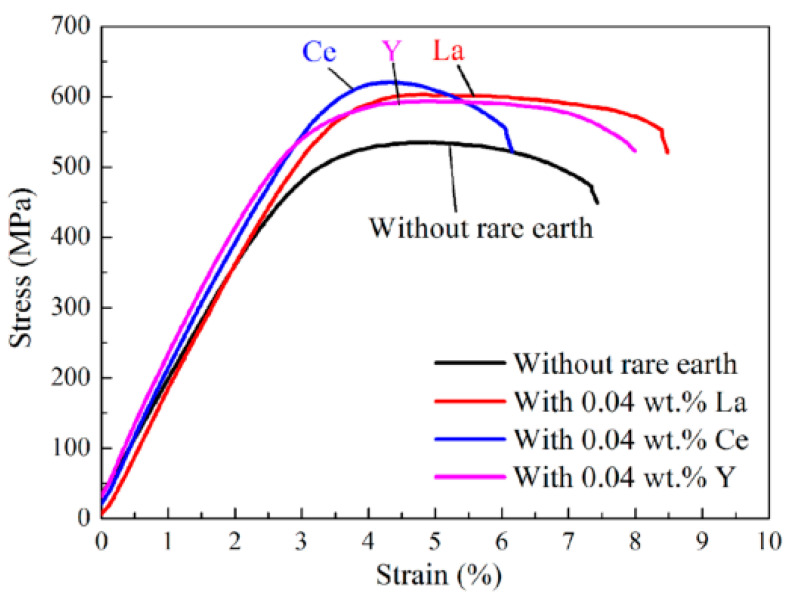
Effects of different rare earths on the tensile strength of (TiB_2p_ + TiB_w_)/Cu composites [36]. Reprinted/adapted with permission from Ref. [36]. Copyright year 2022, Elsevier.

Zou et al. added La to a Cu–TiB_2_ composite to improve the comprehensive properties of the composite and researched the effect of La on the microstructure and mechanical and electrical properties of the Cu–TiB_2_ composite [24]. The results verify that La addition significantly reduces the average size of TiB_2_ particles and promotes the uniform distribution of TiB_2_ particles in copper matrix. With the change of La content, mechanical properties of the composites are improved, as shown in Figure 10 [24]. The mechanisms of La-alloying particle refinement and property improvement were analyzed. An optimal content of 0.04 wt.% La significantly reduced the average particle size of TiB_2_ to 425 nm, a 68% decrease. In addition, the addition of trace La makes TiB_2_ particles more evenly distributed. These two effects are mainly attributed to the introduction of La as a surfactant to reduce the surface tension of the melt [24]. Cao et al. studied the effect of Ce content on the microstructure conductivity and tensile strength of (TiB_2p_ + TiB_w_)/Cu composites. The results verify that an appropriate amount of Ce can significantly refine the grain size of the reinforcement and the copper matrix to improve the morphology and distribution of the reinforcement, as shown in Figure 11 [35]. The tensile strength and electrical conductivity of the composites also change with the content of Ce, but excessive Ce leads to deterioration of the microstructure and the properties of the composites [35]. When Ce content is 0.04 wt.%, the composite has better comprehensive properties, IACS is 78.4% and strength is 671 MPa, which is consistent with the microstructure [35]. Cao et al. reported the effect of rare earth elements on (TiB_2p_ + TiB_w_)/Cu composites. TiB_2_ particles containing rare earth were refined and rounded, and the average particle size was refined from 654 nm (none) to 515 nm (Ce), 567 nm (Y) and 596 nm (La), as shown in Figure 12 [36]. TiB whiskers containing rare earth also showed different thinning effects in diameter and length. After heat treatment and rolling, the tensile strength of the sample increased from 524 MPa (none) to 624 MPa (Ce), 606 MPa (La) and 595 MPa (Y) [36]. Especially at a Ce content of 0.04 wt.%, the tensile strength changes significantly. Microcracks initiate at the interface between the reinforcement and the matrix and then expand along the interface; finally the reinforcement and the matrix are debonded [36]. After Cao et al. added La to a TiB_2_(–TiB)Cu composite, both the composite reinforcement and the copper matrix were refined, and the distribution of reinforcement in the matrix was more uniform [23]. After adding La, the average sizes of TiB_2p_ and TiB_w_ reinforcers were reduced to 0.76 μm and 0.84 μm, decreases of 34.5% and 29.4%, respectively. La addition had no effect on the reinforcement type, but the distribution of reinforcement in matrix was more uniform [23]. The average grain size of copper was reduced from 0.46 μm to 0.33 μm after La was added. The improved the strength of TiB_2p_(–TiB_w_)/Cu–La composites after hot rolling mainly lies in grain refining and Orowan strengthening, as shown in Figure 13 [23].

### 2.3. Micro-Alloying

Rare earth elements in copper matrix composites can form intermetallic compounds with low-melting-point metals in alloys, which can reduce the harm of low-melting-point metals: (1) There is solid solution strengthening when the solution content is given. (2) Reducing the activity of other metals and improving the solubility is conducive to alloying. (3) Rare earth elements can influence the phase-transition mechanism of alloys. (4) Rare earth metals can improve the alloy oxidation film structure and increase the adhesion with matrix to improve the oxidation resistance of alloys [37].

Hao et al. studied the precipitation behavior and strengthening mechanism of Cu–0.4 wt.% Sc alloy during aging [4]. The transition sequence of the precipitated phase in Cu–0.4 wt.% Sc alloy during aging is as follows: supersaturated solid solution → enriched Sc atomic clusters → metastable phase → Cu_4_Sc phase. Cu_4_Sc precipitates in tetractetic lamellar form, the normal plane is parallel to the {111}plane of Cu matrix, and the orientation relationship is (0–22)α//(211)Cu_4_Sc and [011]α//[11–3]Cu_4_Sc evenly distributed in the matrix. In order to improve the electrical and mechanical properties of the alloys, a process combining low-temperature rolling and aging was designed [4]. The electrical conductivity (62.8% IACS) and yield strength (696 MPa) of the alloy were achieved by low-temperature rolling followed by 400 aging for 4 h [4]. The remarkable precipitation strengthening effect of the alloy mainly lies in the extremely small Cu_4_Sc precipitates, which are only 1.5–3 nm in size [4]. The dominant reinforcement mechanisms are coherent reinforcement superposition and Orowan reinforcement. An et al. found that elemental Sc added to a Cu–3 wt.%–Ag–0.3 wt.% Sc system inhibited the formation of a discontinuous Ag-precipitated phase and significantly improved the hardness [38]. Chang et al. studied that the texture composition of as-cast Cu–Ag–Re alloy changed with increased Ce content [15]. With the increase of Ce content from 0.05 wt.% to 1.0 wt.%, the main brass texture changes to copper texture. The ultimate tensile strength and comprehensive mechanical properties of Cu–Ag–Re alloy are 0.2 wt.% Ce and 1.0 wt.% Ce, respectively [15]. Ban et al. reported the influence of Ce on the microstructure and properties of Cu–Ni–Co–Si–Cr alloy [13]. After compression, the texture is mainly <110> fiber, and the texture strength decreases during recrystallization. In addition, under the same conditions, the average orientation φ of Cu–Ni–Co–Si–Cr–Ce (11°) is lower than that of Cu–Ni–Co–Si–Cr (16°) [13]. Liang et al. studied the effects of Y and Zr addition on the microstructure and properties of as-cast Cu–Y–Zr alloy [14]. The results show that, due to the low solid-solubility of Y and Zr in Cu, the amount of precipitated Cu_5_Y and Cu_5_Zr phases increased by adding Y to refine the grains, and thus solid-solution enhancement of Cu–0.5Y–xZr alloy is realized [14]. Saha et al. found that adding elemental Sc to Cu–Zn–Al shape-memory alloy can reduce the phase-transition temperature and thus improve the mobility of the martensite/austenite interface [32]. Sc-free components exhibit the well-known “martensitic stability” characteristic of alloys of the same class, attributed to reduced mobility of the martensitic–austenite interface. However, the addition of Sc tracer significantly reduced the phase-transition temperature, indicating increased fluidity of the martensitic–austenite interface [32]. This reduces the degradation of shape-memory effects (SME) during repeated transformations at lower temperature ranges.

**Figure 14:**
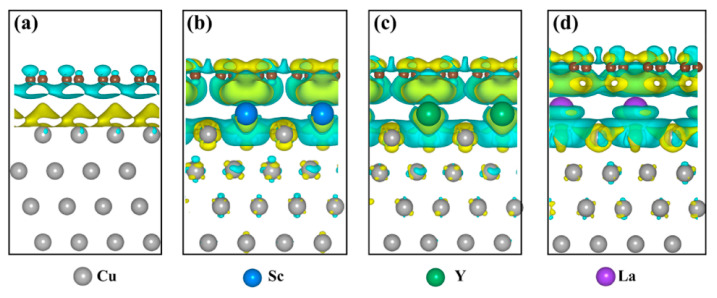
Differential charge density of different interfaces calculated by GGA + vdw: (**a**) clean interface; (**b**) Sc doped interface; (**c**) Y doped interface; (**d**) La doped interface [41]. Reprinted/adapted with permission from Ref. [41]. Copyright year 2022, Elsevier.

**Figure 15:**
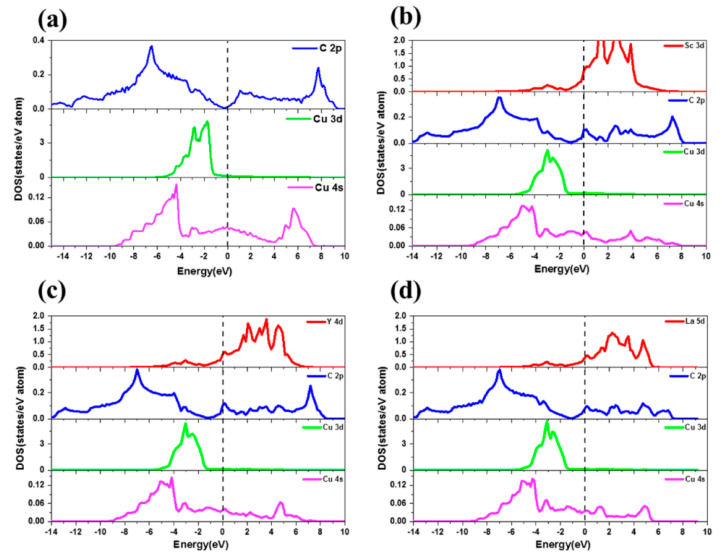
PDOS diagram of Sc–, Y– and La–doped interfaces: (**a**) clean interface; (**b**) Sc–doped interface; (**c**) Y–doped interface; (**d**) La–doped interface [41]. Reprinted/adapted with permission from Ref. [41]. Copyright year 2022, Elsevier.

Zhou et al. studied the enhancement effect of adding a small amount of Dy to GFA and the mechanical properties of (Cu0.47Zr0.45Al0.08)100–xDy [39]. The results show that Dy addition to a Zr–Cu–Al alloy system leads to stronger chemical affinity between Cu–Dy and Al–Dy, thus improving the local stacking efficiency and inhibiting the long-range diffusion of atoms [39]. In addition, the large atomic-size mismatch between Dy and other elements leads to significant distortion of its local atomic environment, which leads to high stability and high GFA of the supercooled liquid [39]. In addition, studies have shown that because Y is chemically active, its affinity for O, N and S is much higher than its affinity for Cu [22,40]. Therefore, it can combine with these elements more readily than with copper and form inclusions in the copper matrix, which will help reduce the solubility of impurities in the copper to maintain the original state of the matrix. Y was added to improve the yield and quality of the synthesized carbon nanotubes as well as the overall performance and properties of the composites. Shi et al. enhanced the weak interfacial bonding of a Cu–graphene (Cu–G) system through rare-earth interfacial doping [41]. In this work, the structural, electronic and mechanical properties of clean and doped Cu–G systems represented by Sc, Y and La were investigated through first-principles calculations [41]. By comparing the interfacial spacing and separation work of the rare-earth doping system with that of the cleaning system, it was verified that the interfacial interaction in the rare-earth doping system was significantly improved [41]. In addition, differential charge density, Bader charge and state density were used to reveal the mechanism of microscopic modification at atomic scale. Then, the fitting function related to element electronegativity was used to obtain the tensile stress–strain curve to determine the quantitative relationship between mechanical properties and interface bonding, providing theoretical guidance for interface modification of Cu–G composites through rare-earth-element doping. Through comparing the separation effects, rare earth elements showed significant improvement to the interface bonding strength of a Cu–G system, with greatest-to-least effectiveness in the order of La > Y > Sc [41]. Differential charge density, Bader charge and state density indicate that rare earth elements enhance interfacial bonding by promoting charge transfer and electron interaction at the interface, as shown in Figure 14 and Figure 15. Rigid tension was realized, and the quantitative relationship between tensile properties and interface bonding was determined by using a fitting function containing electronegativity. The theoretical tensile strength of different interfaces is positively correlated with the separation study. Electronegativity is an important factor affecting mechanical properties and interface bonding of rare-earth doped Cu–graphene systems [41].

## 3. Interface Characteristics

### 3.1. Wettability Mechanism

Rare earth elements can effectively improve the wettability between reinforcement and matrix in copper matrix composites. For one thing, rare earth elements are preferably adsorbed on the grain boundary of the metal matrix and the reinforcement to reduce surface energy, increase interfacial adhesion and reduce wetting angle, thus improving the wettability of the matrix and reinforcement. Further, a direct interfacial reaction can occur, leading to reaction wetting.

Zhang et al. discussed rare earth oxide Sc_2_O_3_ addition to copper/diamond composites [18]. Sc_2_O_3_ formed an orderly and evenly distributed 5–20 nm interfacial chromium carbide layer in the interface region, which played a key role in increasing the bonding of the reaction interface and reducing defects [18]. Sc_2_O_3_ addition increases the number of interfacial heat transfer carriers and makes the interfacial energy system more stable. The diamond/copper interface is thinned by more than 75% to 10 nm–25 nm, and the interface has no defects, which greatly reduces the thermal resistance of the carbide layer [18]. The wettability between copper and diamond particles is effectively improved by the copper/diamond composites. Dang et al. added La_2_O_3_ to a Al–CuO system, and the size, morphology and distribution of in situ-generated Al_2_O_3_ particles changed significantly [5]. The introduction of La_2_O_3_ improved the wettability between CuO and Al, promoted their reaction, accelerated the separation of Al_2_O_3_ and finally resulted in nano–Al_2_O_3_ particles [5]. In addition, La_2_O_3_ weakened the agglomeration-driving force, promoted the uniform distribution of nano–Al_2_O_3_ particles in the matrix, and improved the microstructure of the composites [5]. Li et al. found that the oxidation resistance of Cu–La alloy was mainly due to the interface wrapping of the Cu–La alloy phase between Cu atoms and La_2_O_3_ particles [42]. The influence of the addition of rare earth elements on the oxidation activation energy of Cu–La alloy can be obtained according to the Kissinger equation constructed by Formula (1) [42]:

(1) $$\ln\left(\beta /T_{P}^{2}\right)=\ln\left(AR/E\right)-\left(E/R\right)\cdot \left(1/T_{P}\right)$$

**Figure 16:**
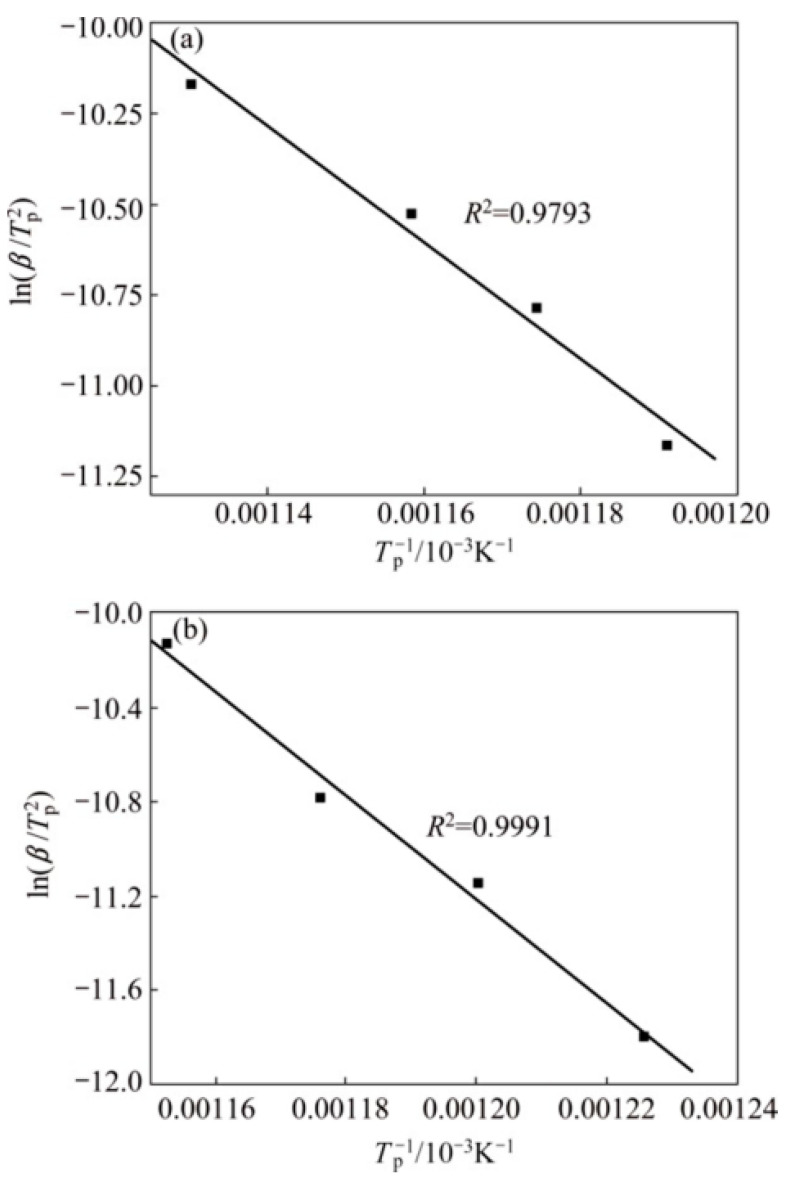
Plots of ln(*β*/*T_p_*^2^) versus 1/*T_p_* of pure Cu and Cu–La alloys: (**a**) pure Cu; (**b**) CuLa alloys [42]. Reprinted/adapted with permission from Ref. [42]. Copyright year 2017, Elsevier.

Thermodynamic calculation shows that La_2_O_3_ is preferentially formed in Cu–La alloy, which is conducive to protection of the Cu matrix, as shown in Figure 16. Kinetic analysis shows that the activation energy of Cu–La alloy is higher than that of pure Cu, indicating that Cu–La alloy has better oxidation resistance.

Carro et al. prepared Cu–1 wt.%Y_2_O_3_ by powder metallurgy and hot isostatic pressure sintering [43]. The results showed that the Y-rich oxides had irregular modifications on grain boundaries, and polygonal Y-rich oxides were dispersed in the copper matrix, so mechanical-property improvement of the alloy was attributed to the existence of enhanced yttrium-rich oxide particles [43]. Huang et al. studied the effect of Y-nanoparticle content in the network on the thermal stability of ultra-fine copper grains [44]. Differences in the thermal expansion coefficients of Y and Cu may produce internal stress in the sintering process, leading to lattice distortion in the copper matrix. As the density of yttrium particles around the dislocation increases, the generated Y_2_O_3_ matrix not only has excellent hardness, abrasion resistance and thermal stability, it also has higher enthalpy than many other reinforced materials, and under the right conditions, it can form a copper crystal orientation relationship at the interface [45]. Compared with the incoherent interface, the coherent interface between the reinforcement and matrix provides both effective strengthening and stronger pinning effect at room temperature, which prevents the growth of copper grains and maintains their initial grain size to delay grain boundary migration at high temperature, ultimately strengthening the matrix material.

### 3.2. Sintering Mechanisms

Rare earth elements can significantly affect the microscopic mechanism of the sintering process based on the following three points: (1) The atomic radii of rare earth elements are larger than that of metal atoms, which leads to distortion of the matrix lattice and increased system energy to keep the lowest free energy, and the irregular arrangement of rare earth atoms towards atoms and enrichment of grain boundaries hinders the free growth of grains. (2) Rare earth elements adsorb other alloying elements and increase the concentration gradient of alloying elements, resulting in local component undercooling and enhanced heterogeneous nucleation of liquid metal matrix. (3) The composition undercooling caused by element segregation results in the formation of segregated compounds and the formation of hetero nucleation sites [3,45,46,47].

Yang et al. found that adding rGO with Ce particles to the Ce–rGO/Cu composite can prevent the obvious coarsening of Cu grains in the sintering process and improve the hardness of the composite [3]. Carro et al. obtained two batches of dispersion-strengthened copper alloys with Cu–0.8 wt.% Y composition using different powder metallurgy methods [46]. They found that Y_2_O_3_ nanoparticles have more potential than Al_2_O_3_ nanoparticles because the driving force of internal oxidation to form Y_2_O_3_ in Cu is slightly higher, the solubility and diffusivity of Y in Cu are lower than that of Al, and the lattice mismatch of Y_2_O_3_/Cu interface is obviously larger than that of Al_2_O_3_/Cu interface [46]. Promoting the formation of Y_2_O_3_ nanoparticles by oxidation and refining the enhanced particle dispersion of powder-particle severe plastic deformation, Y_2_O_3_ was introduced in the sintering process of recrystallization, resulting in the formation of twins. Internal reinforcing particles in the grain boundary and grain have also been found to enhance concrete strength, refining the grain by a pinning effect. Ke et al. prepared copper alloy with Y_2_O_3_ content of 1% by spark plasma sintering (SPS) [47]. The results showed that Y_2_O_3_ particles were evenly distributed in the grain, while dispersing nanoparticles in the grain was an effective method to improve strength and ductility. Because grains can form, fix and therefore accumulate dislocations in grains, pinning dislocation and grain boundaries can effectively refine grains and improve the strength and thermal stability of materials [47]. Yu et al. studied the aging characteristics of Cu–0.6Cr–0.15Zr–0.05Mg–0.02Si alloy containing trace rare earth Y [48]. The results show that the addition of trace Y can effectively increase the nucleation number of precipitate and inhibit the growth of precipitate. Due to its large atomic radius, yttrium is distributed at grain boundaries and dislocations, blocking the short-range diffusion channels of Cr and Zr atoms, but it reduces the diffusion rates of Cr and Zr in copper and inhibits the growth of precipitates such as Cr-rich and Zr-rich particles. The segregation of supersaturated vacancies around Y atoms can reduce the distortion energy and vacancy formation energy, resulting in the formation of vacancy pairs or clusters around Y atoms [48]. These vacancy pairs and clusters gradually gather along the dense row plane to form a solute-rich region, which leads to the increase of nucleation number of precipitates [45]. Ma et al. designed and processed a new Cu–Cr–Yb alloy [49]. The results verified that there was almost no coarsening of the nanoscale chromium-rich precipitates with FCC structure in Cu–Cr–Yb alloy. Thermodynamic analysis showed that the chromium-rich precipitates tended to nucleate with FCC structure [49]. On the one hand, if the content of Yb in chromium-rich nanocrystalline nuclei is high, the nucleation probability is low, and nucleation does not meet the thermodynamic conditions (high nucleation hindrance energy). On the other hand, if the content of Yb in chromium-rich nanocrystalline nuclei is low, the formation of the nuclei does not meet the dynamic conditions due to the large size of the nuclei [49]. Therefore, Yb addition prevents Cr-rich precipitate nucleation and helps to prevent the allographic transformation of FCC structure to BCC structure and Cr-rich precipitate coarsening, thus improving the properties of the alloy.

### 3.3. Solidification Mechanism

Rare earth elements can change the thermodynamic mechanism in the solidification process of copper matrix composites to influence their microstructure and performance: (1) Rare earth elements have strong affinity with oxygen, sulfur, phosphorus, nitrogen and other elements; the standard free formation energy of the product is relatively low. Therefore, if the melting point of the reaction product is higher and the density is lower, then the reaction product can be removed from the alloy and reduce the segregation at the grain boundary. (2) Solidification reactions can reduce the influence of impure elements on the surface tension of the alloy [14,24,36,50,51].

Cao et al. reported the effects of rare earth elements on (TiB_2p_ + TiB_w_)/Cu composites. The effects of rare earth elements on the morphology and size of the reinforcement are mainly due to segregation in the stage of promoting nucleation and inhibiting growth [36]. Liang et al. found that after Y was added, a zirconium-rich phase in the alloy could easily form nucleation during phase precipitation. Cu_5_Zr phase was precipitated in the solidification process of as-cast Cu–0.5Y–0.1Zr alloy, which promoted the formation of Cu_5_Y phase as an inhomogeneous nucleus and finally formed Cu_5_Y and Cu_5_Zr phases [14]. Mao et al. found that rare earth can remove harmful elements such as S and O in Cu–30Ni alloy and refine the as-cast structure, which has an obvious effect on purifying the melting alloy [50]. As the melting temperature of rare earth phase is higher than that of copper and nickel, particles containing rare earth solidified earlier than that of copper and nickel [50]. These pre-solidified particles accelerate dendrite nucleation by providing sufficient nucleation centers and then limit dendrite coarsening by binding to dendrite boundaries during dendrite growth. However, an excess of rare earth tends to delay dendrite boundary movement and promote dendrite growth along the preferred direction. Zhou et al. found that high-La-mixed rare earth has good deoxidation and impurity removal ability and can react with impurities such as magnesium, iron and silicon to form rare earth oxides located at grain boundaries [51]. Therefore, with the addition of 0.01~0.015% La, the solid solubility of the oxide precipitated from the liquid decreases, and the high lanthanum rare earth refines the grain. Rare earth compounds cluster at grain boundaries, so they prevent grain growth and react with impurities such as lead and bismuth in the copper liquid to form compounds with high melting points. These compounds are fine, bulbous and well-distributed in the crystal and limit the growth of grains, thus refining the grains and improving the high-temperature strength. La is a typical surface-active material and one of the typical rare earth elements that has a significant effect on nucleation and grain refinement [24]. As the surface tension of La (729 mN/m) is significantly lower than that of Cu (1349 mN/m), the surface tension of the melt is significantly reduced. According to Equation (1), the total free energy is composed of volume free energy and interface free energy, and the nucleation-critical free energy is sensitive to interface energy, leading to the decrease of critical free energy:

(2) $$\Delta \left(G\right)=V\Delta G_{V}+S\sigma_{sl}$$

Therefore, the size of TiB_2_ critical nuclei is greatly reduced, leading to an increase in the probability of nucleation. Consequently, with the same number of Ti and B atoms, more atomic nucleation will lead to smaller TiB_2_ particle size in the composite. According to Equations (3) and (4), the critical nucleation work of the reinforcer is sensitive to the interface energy, and decreased interface energy will lead to significantly decrease critical nucleation work and increased nucleation rate:

(3) $$\Delta G^{*}=\frac{16\pi \sigma_{sl}^{3}}{3{\left(ƊG_{V}\right)}^{2}}$$

(4) $$N=f_{0}c_{0}exp\left[-\frac{\Delta G^{*}}{KT}\right]$$

**Figure 17:**
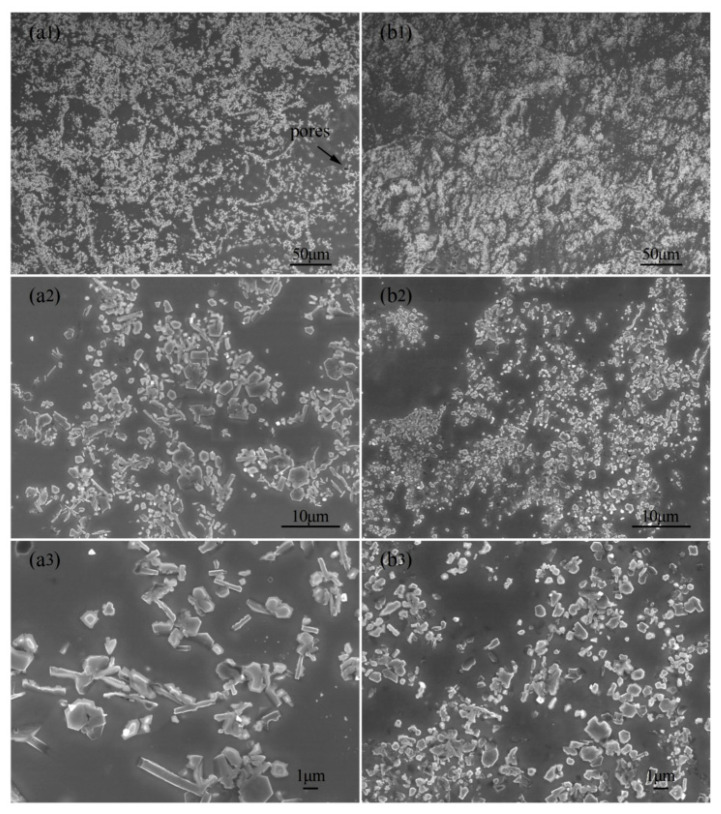
SEM images of as-cast TiB2p(–TiBw)/Cu and TiB2p(–TiBw)/Cu–La composites; (**a1**–**a3**) the as-cast TiB2p(-TiBw)/Cu; (**b1**–**b3**) the as-cast TiB2p(-TiBw)/Cu–La composites [23]. Reprinted/adapted with permission from Ref. [23]. Copyright year 2020, Elsevier.

**Figure 18:**
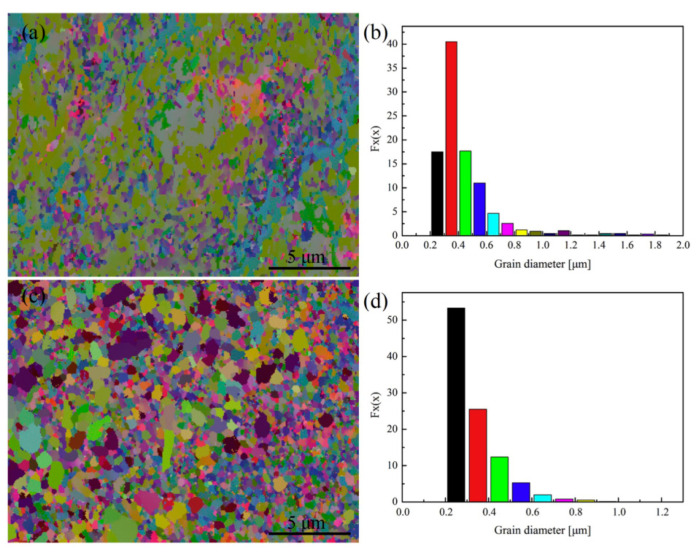
EBSD images and copper grain-size distribution of as-cast TiB2p(–TiBw)/Cu and TiB2p(–TiBw)/Cu–La composites: (**a**,**b**) the as-cast TiB2p(-TiBw)/Cu; (**c**,**d**) the as-cast TiB2p(-TiBw)/Cu–La composites [23]. Reprinted/adapted with permission from Ref. [23]. Copyright year 2020, Elsevier.

**Figure 19:**
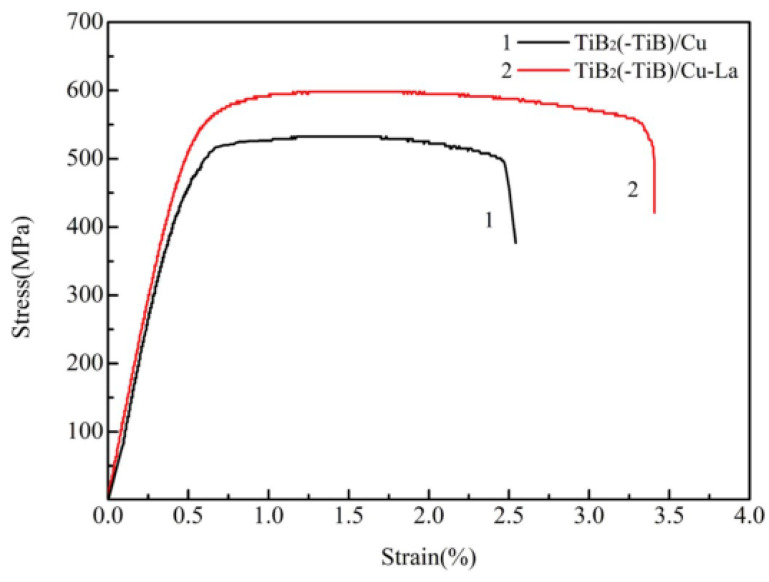
Tensile stress–strain curves of rolled TiB_2p_(–TiB_w_)/Cu and TiB_2p_(–TiB_w_)/Cu–La composites [23]. Reprinted/adapted with permission from Ref. [23]. Copyright year 2020, Elsevier.

La was added to the composite as a typical surface-active element [23]. La addition reduces the critical nucleation work of the reinforcers, leading to the easy nucleation of TiB_w_ and TiB_2p_; that is, the nucleation rate increases. At the same time, the addition of La has a mitigating effect on the further migration and binding of grain boundaries of the reinforcer, which will hinder the growth of the reinforcer and eventually lead to refinement [23]. After La is added, the wettability between the copper melt and the reinforcement is better, and the surface tension is reduced. On this basis, the natural convection formed in the copper melt can improve the penetration rate of the liquid into the void between the reinforcement. Finally, the agglomeration of reinforcement is inhibited, and the reinforcement is evenly distributed in the copper matrix, as shown in Figure 17 [23]. In addition, the grain refinement of copper can be explained from the following three aspects: Firstly, the refined TiB_2p_ and TiB_w_ cannot only serve as effective heterogeneous nucleation sites for copper grains, but also aggregate at grain boundaries to inhibit grain growth. Secondly, the enrichment of La is conducive to the formation of a structural undercooling zone at the front of the S/L interface, which increases the nucleation rate. Finally, La reacts with impurities in the copper melt to form compounds with high melting points, which can prevent the growth of copper grains, which can be seen in the grain-size distribution of the composite in Figure 18 [23]. The refined matrix can effectively improve the mechanical properties of the composites, as shown in Figure 19 [23].

## 4. Strengthening Mechanism

Strengthening mechanisms in metal matrix composites mainly include load transfer, fine-grain strengthening, Orowan strengthening, thermal mismatch strengthening, etc., as shown in Formula (5) [23,24]:

(5) σc=σm+Δσgf+((ΔσOrowan)2+(ΔσCTE)2)12+ΔσLT

The calculation formula of thermal mismatch strengthening is as follows [17]:

(6) $$\Delta \sigma_{CTE}=\alpha \mu b\sqrt{\rho }$$

Thermal mismatch strengthening is attributed to the material system being subjected to rapid heating or cooling conditions, whereas the heating and cooling rates in common preparation processes are generally slow, and the strengthening mechanism of load transfer is rare in particle strengthening. Therefore, in the system of copper matrix composites reinforced by rare earth elements, the above enhancement effect can be ignored [34]. The symbol $\sigma_{m}$ represents the strength of the matrix itself. The Hall–Patch effect caused by grain refinement and Orowan strengthening caused by dispersion distribution of reinforcement are discussed [15,16].

### 4.1. Grain Boundary Strengthening

**Figure 20:**
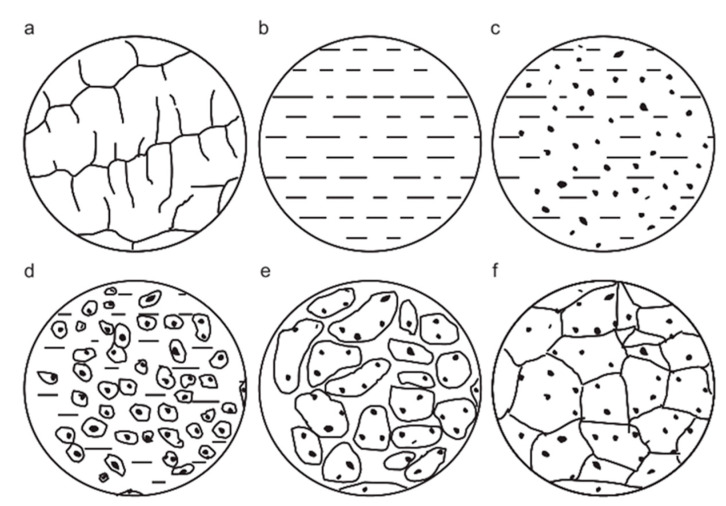
Schematic diagram of solidification: (**a**) original microstructure; (**b**) melts; (**c**) in situ reaction; (**d**) nucleation; (**e**) growth and aggregation; (**f**) ultimate microstructure [12]. Reprinted/adapted with permission from Ref. [12]. Copyright year 2013, Elsevier.

Zhuo et al. studied the strengthening mechanism of Y_2_O_3_ addition to Cu-matrix composites and found that the volume fraction of Y_2_O_3_ produced by 0.4 wt.% Y in the alloy is about 0.9 vol.% [12]. Compared with as-cast Cu, eutectic clusters and dendrites have been replaced by equiaxed grains with an average grain size of 150 μm [12]. During the solidification process of molten Cu–Y alloy, structure transformation occurs with the in situ reaction of Y_2_O_3_ particles. The detailed process is shown in Figure 20. When no oxygen atoms enter a Cu–Y melt, due to the dynamic equilibrium between solidification and melting at liquidus temperatures, the melt cannot crystallize. However, when oxygen atoms in the atmosphere diffuse into the melt, Y is oxidized first, forming Y_2_O_3_ particles. Therefore, the local Y content in the area around Y_2_O_3_ particles will decrease and then gradually approach zero [12]. In addition, Y_2_O_3_ particles can also be used as nucleating agents to further promote solidification. The combination of these two effects leads to isothermal solidification of the Cu–Y melt and eventually to equiaxed grain structure [12].

According to the Hall–Patch formula, grain refinement can cause strengthening in composites [16,27]:

(7) $$\Delta \sigma_{gf}=k\left(d_{c}^{-0.5}-d_{m}^{-0.5}\right)$$

(8) $$\sigma_{yield}=\sigma_{0}+\frac{k_{y}}{\sqrt{d}}$$

where $d_{c}$ and $d_{m}$ are the grain size of composite material and pure metal, respectively. It is not difficult to see that the smaller the grain size of composite material, the more obvious the enhancement effect of reinforcement [5,23].

### 4.2. Dispersion Strengthening

Maharana et al. electrodeposited Cu–Y_2_O_3_ composite coating on copper substrate with Y_2_O_3_ ultrafine particles of different concentrations (10 and 30 g/L) as raw materials, and found that the coating had better surface mechanical properties and oxidation properties than pure copper [52]. After the addition of Y_2_O_3_, the oxide dispersion serves as the heterogeneous nucleation center of copper, making the microstructure of Cu finer. Therefore, ultrafine Y_2_O_3_ particle addition to the sedimentary layer will affect the competitive formation and crystal growth of copper nuclei, which means that there are more nucleation sites for copper ions [52]. Avettand-Fènoëlto et al. utilized Y_2_O_3_ particles with copper to produce oxide-dispersion-reinforced materials; with the disintegration of the initial Y clusters, the fragmentation of elemental Y particles and the homogenization of the second phase are dispersed [53]. The ultimate tensile strength (UTS) of Cu–0.9Y_2_O_3_ composite prepared by Zhou is 568 MPa [12]. Because at room temperature the oxide ceramic particles are usually non-deformed—that is, these particles are difficult to be cut by dislocation based on a dislocation loop mechanism—Orowan strengthening is considered to be the only factor affecting the particle-strengthening mechanism [12]. In addition, the nano-sized Y_2_O_3_ particles have a coherent match with the copper matrix, indicating that the shear mechanism also contributes to the increase of strength. The feasibility of a shear-strengthening mechanism depends on the size of Y_2_O_3_ particles. Some Y_2_O_3_ particles will be sheared by dislocations during deformation, and the shear-strengthening mechanism is conducive to the improvement of strength [12]. The analysis shows that Orowan mechanism and shear mechanism are the main reinforcing mechanisms. This strengthening is attributed to the enhanced dislocation interaction caused by the particle Orowan detour.

Orowan strengthening works when the reinforcement is dispersed in the matrix. Therefore, the reason why the enhancement phase reunion cannot enhance effectively is explained [15,16]. The tightly bonded semi-coherent interface matching between dispersed nanoparticles and matrix ensures good dislocation resistance, thus realizing Orovan strengthening [27]:

(9) ΔσOrowan=0.4MGbπ(1−v)12ln(d¯/b)λ¯

(10) $$\bar{d}=\sqrt{2/3}d$$

(11) $$\bar{\lambda }=\left(\sqrt{\pi /\left(4f\right)-1}\right)\bar{d}$$

According to the Orowan formula, the shear modulus of the matrix, grain size and volume fraction of reinforcement are closely related to Orowan strengthening. Precipitation strengthening is controlled by an Orowan dislocation bypass mechanism or dislocation shearing mechanism, and the mechanisms lead to small strength increments [23]. In general, when the radius of the precipitate exceeds the critical value or the precipitate is irrelevant to the matrix, the Orowan dislocation bypass mechanism occurs [5].

### 4.3. Hybrid Reinforced

Two or more rare earth compounds can be co-doped in copper matrix composites; the existing forms and effects of co-doped rare earth elements in copper matrix need to be further studied. Adding a single strengthening phase around the matrix easily produces agglomeration, and the stress concentration around the agglomeration-strengthening phase will destroy the mechanical properties of the matrix [41]. Adding a variety of rare earth oxides to achieve composite strengthening is beneficial to improve the mechanical properties of the material [14,50,54,55].

Liang et al. studied microstructure and properties of as-cast Cu–Y–Zr alloy with Y and Zr addition [14]. The results show that the precipitated Cu_5_Y and Cu_5_Zr phases could refine the grains, and thus solid solution enhancement of Cu–0.5Y–xZr alloy was realized [14]. Mao et al. prepared Cu–30Ni–xRE (x = 0–0.213) alloy by metal mold casting, in which RE composition was 30–La–45Ce–13Pr–12Nd–1Fe–0.1Mg–0.Pb–0.05Zn–0.05C [50]. The results show that rare earth elements can refine the dendrite structure and grain size of Cu–30Ni alloy and purify the alloy. In addition, rare earth can form the second phase with other elements and play the role of strengthening the second phase, and can purify grain boundaries, refine the structure, and remove impurities and slag to improve the ductility of Cu–30Ni alloy [50]. Rare earth intermetallic compounds have high strength, high hardness and excellent high-temperature performance and oxidation resistance [14,41]. As metal phases, they have a close thermal expansion coefficient with copper matrix and better interface bonding ability compared than ceramic phases [14]. Using rare earth intermetallic compounds as reinforcement of copper matrix composites can greatly improve mechanical properties and oxidation resistance of copper matrix composites at high temperatures so that they can meet harsher service conditions.

## 5. Conclusions and Outlook

Because the action mechanism of rare earth elements (La, Ce, Y, Dy, Sc, etc.) on copper matrix composites is closely related to their thermodynamic characteristics, by studying the interaction mechanism between rare earth elements and the constituent elements of copper matrix composites, rare earth elements are widely used in the preparation of copper matrix composites and improve their properties due to their unique characteristics. The strengthening methods and mechanisms of rare earth elements and their oxide-reinforced Cu matrix composites mainly include the following aspects.

Compared with Cu matrix, rare earth elements react more easily with harmful impurities in the matrix to produce oxides or intermetallic compounds, making them evenly distributed in the matrix while purifying the gain interface. The second phase in the matrix can control the uniformity of microstructure and significantly hinder grain boundary movement and grain growth so that composites with fine grain size can achieve fine grain strengthening effect. Second, there is usually a large atomic size mismatch between rare earth elements and other elements, which leads to significant distortion of local atoms, resulting in lattice distortion. This has a strong blocking effect on dislocation movement, and the growth of grains is inhibited by the pinning effect of dislocations to refine the grains. Therefore, fine grain strengthening by inhibiting grain growth and dispersion strengthening by the second phase formation are the main ways rare earth elements strengthen Cu matrix composites.

For Cu matrix composites, the strengthening mechanisms of rare earth elements is mainly grain boundary strengthening and dispersion strengthening under Orowan mechanism by affecting interface wettability, composite sintering and nucleation solidification. Rare earth elements can be adsorbed on the grain boundary of the matrix and reinforcement to reduce the surface energy, increase the interfacial adhesion work and reduce the wettability angle, thus improving the wettability of the matrix and the reinforcement to improve the microstructure of the matrix. The atomic radius of rare earth elements is larger than the radius of metal atoms; thus, the irregular arrangement of rare earth atoms toward atoms and enrichment of grain boundaries hinder the free growth of grains due to the distortion of matrix lattice, which can increase system energy to keep the lowest free energy. Rare earth elements adsorb other alloying elements and increase the concentration gradient of alloying elements to enhance heterogeneous nucleation of liquid metal matrix. As rare earth elements have strong affinity with oxygen, sulfur, phosphorus, nitrogen and other elements, the standard free formation energy of the product is relatively low; therefore, solidification reactions can reduce the influence of impure elements on the surface tension of the alloy to realize the performance optimization of Cu matrix composites.

To sum up, at present, rare earth elements are mainly used as microalloying elements to enhance Cu matrix composites, and proper addition to the matrix can effectively regulate the microstructure of composite materials, improving the performance. Existing research based on rare earth elements as auxiliary elements show that excessive addition of rare earth elements leads to interface agglomeration and handicaps performance. High-performance copper alloys based on rare earth elements can be developed, and the influence mechanism and effects of rare earth elements cooperating with other alloy elements to improve the properties of copper matrix composites can be deeply analyzed and discussed. At present, the research on rare-earth-element-reinforced Cu matrix composites is mainly through the addition of single rare earth elements, but research on multi-rare-earth-element mixed enhancement or rare-earth-element-mixed traditional enhancement is less. In addition, due to the different reaction products of different rare earth elements in the matrix, the mechanical, thermal and electrical properties of the generated phase are also greatly different. Therefore, in order to meet the application of different service environments in the engineering field, it is necessary to increase the research on the influence of rare earth elements on the properties of composite materials in various aspects to select appropriate reinforcement methods. At the same time, in addition to the enhancement of Cu matrix composites, their unique characteristics can be widely used in the preparation and performance improvement of metal matrix composites overall, maximizing the use of rare earth elements.

## Data Availability

Not applicable.
